# 93132 Relationship between Level of Response to Alcohol and Body Weight Status in Individuals across the Spectrum of Alcohol Use and Misuse

**DOI:** 10.1017/cts.2021.750

**Published:** 2021-03-30

**Authors:** Rhianna R. Vergeer, Bethany L. Stangl, Melanie L. Schwandt, Nancy Diazgranados, Vijay A. Ramchandani

**Affiliations:** 1 Laboratory on Human Psychopharmacology; 2 Division of Intramural Clinical and Biological Research; 3 National Institute on Alcohol Abuse and Alcoholism (NIAAA)

## Abstract

ABSTRACT IMPACT: Improved understanding of the relationship between level of response to alcohol and body weight in the context of other contributing factors will help inform prevention and intervention efforts regarding obesity. OBJECTIVES/GOALS: We evaluated the association between level of response to alcohol and weight status across the spectrum of alcohol misuse. We hypothesized that lower level of response to alcohol would be associated with heavier weight, after controlling for obesity risk factors like food addiction, impulsivity, and low socioeconomic status. METHODS/STUDY POPULATION: Adult participants (N=587) enrolled in NIAAA’s natural history study completed Self-Rating of the Effects of Alcohol (SRE), a retrospective measure of level of response to alcohol, along with Lifetime Drinking History (LDH); Yale Food Addiction (FA) Scale; Barratt Impulsiveness Scale (BIS); Delayed Discounting Task (DDT). Structured Clinical Interviews for DSM disorders were conducted to identify individuals with current alcohol use disorder (AUD). Body mass index (BMI), computed from height and weight measured during the study, and used to stratify participants into 3 groups: normal weight (N=222), overweight (N=219), or obese (N=146). RESULTS/ANTICIPATED RESULTS: SRE scores during heaviest drinking period were lowest in the heavier weight group, after accounting for FA, impulsivity, alcohol-related, and demographic variables (ê•2=238.5, p=0.002, Cox and Snell Pseudo R2=0.43). Compared to the obese group, normal weight and overweight groups had fewer FA symptoms and higher BIS cognitive complexity (p values<0.01) but similar rates of current AUD. Relative to the obese group, the normal weight group was more likely to be White, and to have lower household incomes but more education, more years of heavy drinking (LDH), and steeper delayed discounting, p values ≤0.03. The overweight group had a higher proportion of males than did the obese group, p<0.001. DISCUSSION/SIGNIFICANCE OF FINDINGS: Lower level of response during heaviest drinking period was significantly associated with current weight status, suggesting a relationship between alcohol sensitivity and BMI. Future work will explore pharmacokinetic-pharmacodynamic and additional risk factors underlying this relationship.

